# Application of the Composite Quality Score (CQS-2B) versus Cochrane’s Risk of Bias tool (Version 2) in systematic reviews of clinical trials – an exploratory study

**DOI:** 10.3389/fmed.2024.1307815

**Published:** 2024-05-02

**Authors:** Steffen Mickenautsch, Stefan Rupf, Veerasamy Yengopal

**Affiliations:** ^1^Review Centre for Health Science Research, Johannesburg, South Africa; ^2^Department of Community Dentistry, School of Oral Health Sciences, Faculty of Health Sciences, University of the Witwatersrand, Johannesburg, South Africa; ^3^Faculty of Dentistry, University of the Western Cape, Cape Town, South Africa; ^4^Synoptic Dentistry, Saarland University, Homburg, Germany

**Keywords:** Composite Quality Score, systematic review, trial appraisal, clinical trial, practical application

## Abstract

**Objectives:**

To explore whether systematic review conclusions generated from Cochrane’s second version of its Risk of Bias tool (RoB 2) for trial appraisal differ when the Composite Quality Score, Version 2.B (CQS-2B) is used instead and to develop a testable hypothesis based on these findings.

**Methods:**

PubMed was searched for one single systematic review. From the review’s accepted trials, data concerning effect estimates and overall bias risk according to the RoB 2 tool were extracted. All trial reports were appraised again using the CQS-2B. Datasets were stratified according to overall bias risk (RoB 2) or corroboration (C-) level (CQS-2B). The effect estimates from trials with ‘low bias risk’ (RoB 2) and with highest C-level (CQS-2B) were pooled separately. These pooled effect estimates were statistically and all clinical conclusions qualitatively compared.

**Results:**

The pooled effect estimates for trials with ‘low bias risk’ (RoB 2) were −0.07, 95% CI: −0.10 to −0.04 (*I*^2^ = 0.0%) and for the highest C-levels (CQS-2B) 0.08, 95% CI: −0.12 to −0.04 (*I*^2^ = 57.0%). The difference was statistically not significant (*p* = 0.70). Contrary to the RoB 2 tool, no clinical conclusions in line with the CQS-2B were made, because the effect estimates were judged to be erroneously overestimated, due to high risk of bias.

**Conclusion:**

A testable hypothesis was generated suggesting that trial appraisal using the CQS-2B may provide more conservative conclusions based on similar data than with the RoB 2 tool.

## 1 Introduction

The Composite Quality Score (CQS) has recently been developed as an appraisal tool for prospective, controlled, clinical therapy trials (1). Its current version (CQS-2B) comprises of four trial appraisal criteria that are related to the random allocation of subjects to treatment groups, the concealment of the random allocation, the process of double-blinding and a minimum required sample size limit (2, 3). These criteria are presented in Table 1.

**Table 1:** CQS-2B appraisal criteria.

Criterion I	‘Randomisation’ for allocation to treatment groups is in some form reported in the text
Criterion II	Any assurance that the patient allocation to treatment groups according to the random sequence was applied by an independent agent or agency, not otherwise involved in the trial, is in some form reported in the text
Criterion III	Double-blinding or the blinding of at least two out of the three groups: trial participants trial personnel and trial outcome assessors in some form reported in the text
Criterion IV	The sample size of any particular treatment group reported in the trial is not less than *N* = 100

The application of the CQS-2B includes binary trial report rating per appraisal criterion (Scores: 0 = No/invalid/falsified, 1 = Yes/corroborated); multiplication of all scores to an overall appraisal score, and identification of invalid trial reports on basis of a zero overall appraisal score.

During the application of the CQS-2B several corroboration (C-) levels can be recognized. These C-levels indicate how many consecutive criteria a trial has complied with (e.g., level C2 indicates compliance with Criterion I and II, etc.). A C-level for a trial is reached before one criterion is 0-score rated, e.g., level C2: Criterion I and II = 1-score, Criterion III = 0-score, whereafter the C-level remains the same even if a following criterion is 1-score rated (1).

The CQS-2B follows the understanding that any characteristic of a trial related to any form of error outside of any applied trial appraisal criteria set may invalidate the trial’s results. Hence, no certainty that a trial is of ‘low bias risk’ is ascribed to an overall 1-score rating. Such overall 1-score rating only suggests that a trial is ‘corroborated.’ This means that no evidence for high bias risk was found. It does not suggest that such evidence may not be established during future appraisals with any additional appraisal criterion (1).

These premises are in keeping with the deductive falsification approach, which states that although ‘low bias risk’ cannot be established, it is always possible to establish whether bias risk is high (4). ‘High bias risk’ is present when trial characteristics that are essential for reflecting the true effect estimate are absent or insufficiently applied (1).

All CQS-2B criteria describe trial characteristics whose absence or insufficient application is associated with a systematic diversion from the true effect estimate. Criterion I is based on the results of a Cochrane systematic review of meta-epidemiological studies that showed that results from non-randomized trials differ from that of randomized trials (5). A further systematic review of meta-epidemiological studies (2) established a statistically significant effect of over-estimation associated with trials when allocation concealment (Criterion II) and double-blinding (Criterion III) are absent or uncertain and where the sample size is below 100 per intervention group (Criterion IV).

In addition, application of the Composite Quality Score (version: CQS-2) has been associated with a very high inter-rater reliability. During an inter-rater reliability study, four independent raters appraised 45 trial reports from 16 different clinical specialities (6). All raters had slight content knowledge about the rated trials and no extensive expertise in the conducting systematic reviews of randomized controlled trials. They only received the study-protocol for information about how to apply the CQS-2 and no calibration nor training for using the CQS-2 was carried out. However, an almost perfect inter-rater agreement in line with the Landis/Koch Kappa’s Benchmark Scale (7) (Brennan-Prediger coefficient 1.00; 95% CI: 0.94–1.00) was achieved (6).

Against this background (2–5), the CQS-2B may be considered a viable alternative to the RoB 2 tool. It has been established that the RoB 2 tool is associated with poor inter-rater reliability (Fleiss’ Kappa 0.16; 95% CI: 0.08–0.24) (8) and its application found to be complex and demanding. The tool further requires formal training and pilot runs prior to its application. In addition, integrated teamwork, a high expertise in the systematic review’s subject matter, in clinical epidemiology, as well as in trial methodology and statistics, are needed (8).

Such complexity and the poor inter-rater reliability of the RoB 2 tool stands in contrast to the worldwide continuously increasing volume of clinical trials (9) and the need for faster, less complicated, but effective, reliable trial appraisal.

Against this background, the CQS-2B may offer an alternative. However, this raises the question of whether the application of the CQS-2B, instead of the second version of Cochrane’s Risk of Bias tool, would generate different systematic review conclusions. Because the applicability and validity of the CQS-2B has not previously been investigated, the aim of this study was to explore whether systematic review conclusions originally based on the RoB 2 tool do not differ when the CQS-2B is used instead and to use its findings to develop a testable hypothesis for further research.

## 2 Methods

This study investigates a research question that has not previously been studied in depth. It has therefore adopted an exploratory nature, designed to establish a first preliminary understanding about the topic and to generate a working hypothesis, for testing at a later stage. All study methods were pre-specified in a protocol and made available online prior to the start of the study (10).

### 2.1 Literature search

PubMed was searched up to 15th of February 2023 for one systematic review report using the search term: “cochrane risk of bias tool 2,” including the limits – article type: ‘systematic review’ and ‘free full text’; sorted by: ‘publication date.’ One reviewer (SM) conducted the search by screening all abstracts online. The first systematic review report was selected that complied with all the following criteria:

- At least 20 prospective, clinical, controlled therapy trials included into meta-analyses;
- Computable datasets for test and control group reported (for dichotomous data: number of events, total number of subjects; for continuous data: total number of subjects, mean values with standard deviation (SD) or standard error (SE));
- Trial appraisal using the RoB 2 tool and reporting of the overall appraisal decision per trial concerning bias risk (‘high risk’; ‘low risk’; ‘some concerns’);
- Inclusion of at least 5 trials in at least one single comparison per measured outcome;
- Publication language: English.

A second reviewer (SR) double-checked whether the selected systematic review report complied with the listed set of criteria. Any arising discrepancies were resolved by discussion and consensus.

### 2.2 Data extraction

All trial reports traced in full copy and the following trial information extracted:

- Full reference details;
- Overall appraisal decision based on the RoB 2 tool;
- Computable data.

One reviewer (SM) extracted all information and entered them into an MS Excel file. A second reviewer (SR) double-checked all extracted data and corrected possible errors.

### 2.3 Trial re-appraisal

All trial reports were re-appraised using the CQS-2B for potential bias risk. For each awarded 1-score per CQS-2B criterion, the supporting verbatim quotes were extracted from the appraised trial report and entered into a verbatim table.

Two reviewers (SM, SR) appraised each trial independently. Any discrepancies in the review outcome were resolved by discussion and consensus. The result for each criterion was entered into an appraisal table and the corroboration levels established per trial.

### 2.4 Data analysis

In line with published recommendations for Cochrane’s RoB 2 tool (11), the extracted datasets for all comparisons per measured outcome were stratified by overall bias risk according to the RoB 2 tool and by corroboration level according to the CQS-2B. For each appraisal tool, the stratified trial data for any comparison per measured outcome were statistically pooled by use of the standard Mantel–Haenszel statistics with a random-effects model.

All pooled ‘low bias risk’ effect estimates (RoB 2) and all pooled effect estimates of the highest C-level with data (CQS-2B) for all comparisons for each measured outcome were in turn pooled by use of a random effects meta-analysis, separately for each appraisal tool. DerSimonian and Laird’s method of moments estimator was used to estimate the variance (12). Statistical inconsistency was quantified by use of the I^2^ statistic (13) and the two resulting pooled effect estimates for RoB 2 and the CQS-2B were statistically compared by use of the Wald-test. The null-hypothesis was tested that both are not significantly different. A 5% significance level was used.

In addition, for all comparisons per measured outcomes, clinical conclusions that followed from the pooled ‘low bias risk’ estimates (RoB 2) as well as the highest C-level (CQS-2B) were qualitatively compared by use of a comparison table.

## 3 Results

The literature search yielded 91 citations. From these, the systematic review of clinical trials by Sellem et al. (14) concerning the impact of replacing individual dietary saturated fatty acids (SFA) on cardio-metabolic health biomarkers, was the first of the generated citation list that complied with all the selection criteria and thus was selected for our study (14).

This systematic review accepted a total of 34 clinical trials for quantitative synthesis and reported results of four comparisons [palmitic acid vs. monounsaturated/polyunsaturated fatty acid (PUFA/MUFA); palmitic acid vs. stearic acid; palmitic acid vs. oleic acid, and stearic acid vs. PUFA/MUFA] with meta-analyses for six outcome measures [effect of dietary fat substitutions on LDL, HDL, total cholesterol, triacylglycerol, apoA-I concentrations and apoB concentrations (Supplementary material 1/Sheet 1)]. Each comparison included 18, 5, 9 and 4 trials, respectively. Two trials provided datasets for more than one comparison. Since the number of trials for the comparison ‘stearic acid vs. PUFA/MUFA’ was <5, the data of this comparison were not included for this study.

All trials were re-appraised by use of the CQS-2B. Thirty-two (32) trials were rated with an overall 1-score at C1-level, one trial at C2-level and one trial at C3-level. None of the 34 appraised trials were rated with an overall 1-score at C4-level (Supplementary material 2).

The extracted datasets for all comparisons per measured outcome were stratified, as well as statistically pooled according to overall bias risk (RoB 2) and corroboration level (CQS-2B) and are reported in Supplementary material 1/Sheets 2–4. Stratification for ‘low risk of bias’ according to the RoB 2 tool yielded three trials for all comparisons and outcome measures. Stratification according to the CQS-2B yielded one trial at C3-level for the palmitic acid vs. stearic acid comparison, one trial at C2- level for the palmitic acid vs. MUFA/PUFA comparison and nine trials at C1-level for the palmitic acid vs. oleic acid comparison as highest corroboration levels with data.

**Figure 1:**
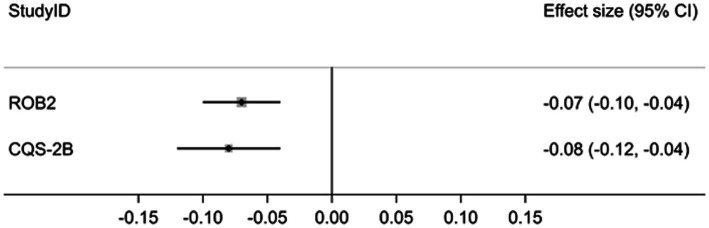
Forest plot of pooled effect estimates.

The pooled effect estimates across all comparisons and measured outcomes for trials with ‘low bias risk’ (RoB 2) was −0.07, 95% CI: −0.10 to −0.04 (*I*^2^ = 0.0%) and for the highest C-levels (CQS-2B) was 0.08, 95% CI: −0.12 to −0.04 (*I*^2^ = 57.0%) (Figure 1).

The difference between both estimates was statistically not significant (*p* = 0.70) and the null-hypothesis was accepted (Supplementary material 1/Sheet 5).

Details of the qualitative comparison between the clinical conclusions established from the results of trials with ‘low bias risk’ (RoB 2) and of trials with highest C-levels (CQS-2B), per clinical comparison type and measured outcome, are shown in Supplementary material 3 and a summary of the results is presented in Table 2.

**Table 2:** Qualitative comparison summary.

Comparison	Measured outcome	Clinical conclusion	
		RoB 2 tool*	CQS-2B**
Palmitic acid vs. MUFAs+PUFAs (18 Trials)	Effect of dietary fat substitutions on LDL-C	Has no beneficial effect	No conclusion. There is a high risk that the established effect estimate is overestimated due to lack of double blinding.
	Effect of dietary fat substitutions on TC	**Has beneficial effect**	
	Effect of dietary fat substitutions on HDL-C	**Has beneficial effect**	
	Effect of dietary fat substitutions on triacylglycerol	Has no beneficial effect	
	Effect of dietary fat substitutions on apoA-I concentrations	Has no beneficial effect	
	Effect of dietary fat substitutions on apoB concentrations	**Has beneficial effect**	
Palmitic acid vs. Stearic acid (5 Trials)	Effect of dietary fat substitutions on LDL-C	Has no beneficial effect	No conclusion. There is a high risk that the established effect estimate is overestimated due to too low sample size.
	Effect of dietary fat substitutions on TC		
	Effect of dietary fat substitutions on HDL-C		
	Effect of dietary fat substitutions on triacylglycerol		
Palmitic acid vs. Oleic acid (9 Trials)	Effect of dietary fat substitutions on LDL-C	Has no beneficial effect	No conclusion. There is a high risk that the established effect estimate is overestimated due to lack of allocation concealment.
	Effect of dietary fat substitutions on TC		
	Effect of dietary fat substitutions on HDL-C		
	Effect of dietary fat substitutions on triacylglycerol		
	Effect of dietary fat substitutions on apoA-I concentrations		
	Effect of dietary fat substitutions on apoB concentrations		

C, Cholesterol; TC, Total cholesterol; RoB, Risk of bias tool; CQS, Composite quality score; MUFA, Monounsaturated fatty acid; PUFA, Polyunsaturated fatty acid. *From low bias risk trials. **From Trials with highest Corroboration level.

From ‘low bias risk’ trial results (RoB 2), it was concluded that replacement of palmitic acid with MUFAs+PUFAs had a beneficial effect on the fasting total and HDL cholesterol concentrations, as well as on the apoB concentrations. No benefit was deduced from the results of all other comparisons.

From the results of the highest C-level trials (CQS-2B), no clinical conclusion as to any beneficial effect or lack thereof were made, due to the high risk that the established effect estimates were overestimated. Such overestimation was ascribed to lack of blinding (palmitic acid vs. MUFAs+PUFAs), too low sample size (palmitic acid vs. stearic acid) and lack of allocation concealment (palmitic acid vs. oleic acid).

## 4 Discussion

### 4.1 Study results

The results of this exploratory study suggest that trial appraisal using the CQS-2B after stratification according to highest corroboration level with data yields not statistically significantly different effect estimates than trial appraisal using Cochrane’s RoB 2 tool after stratification according to lowest bias risk. The pooled point estimates and confidence intervals for both tools suggest similar effect magnitude and effect direction (Figure 1). In contrast, inter-study heterogeneity was low with the RoB 2 tool (*I*^2^ = 0.0%; *p* = 0.578) but statistically significant for the CQS-2B (*I*^2^ = 57.0; *p* = 0.003) (Supplementary material 1/Sheet 5). This difference may be explained on basis that for the latter tool trial data were pooled from several different corroboration levels (C1–3), while for the former all data were extracted from trials with the same ‘low-bias risk’ status.

Qualitative comparison between both tools showed that the interpretation of the similar effect estimates in line with the CQS-2B yielded far more conservative conclusions than when interpreted in line with the RoB 2 tool (Table 2). During trial appraisal using the RoB 2 tool, no distinction was made in terms of further potentials in bias risk or systematic error for trials that were already rated as of ‘low-bias risk.’ Hence, all single effect estimates for trials rated of ‘low risk’ were used to deduce a clinical conclusion. These included that dietary substitution of palmitic acid (the most common saturated fatty acid in the human diet) with a combination of monounsaturated and polyunsaturated fatty acids (MUFA/PUFA) has a beneficial effect on the fasting HDL and total cholesterol concentrations, as well as on apoB concentrations. No beneficial effect was deduced from effect estimates of all other comparisons and measured outcomes (Supplementary material 3).

In contrast, trial appraisal using the CQS-2B did not identify any trials that complied with all its four appraisal criteria (C4-level). Hence, no clinical conclusions as to the benefit or the lack thereof were deduced from the established effect estimates. One trial was rated as C3-level for the comparison of palmitic acid vs. stearic acid; one trial was rated as C2-level for the comparison of palmitic acid vs. MUFAs and PUFAs and nine trials were rated as C1-level for the comparison of palmitic acid vs. oleic acid (Supplementary material 3). Accordingly, a high risk was assumed that all established effect estimates were overestimated, due to a too low sample size (Criterion IV = 0-score), lack of double-blinding (Criterion III = 0-score) and lack of allocation concealment (Criterion II = 0-score), respectively. The assumptions for such high-bias risk were made on the basis of empirical evidence from a systematic review of meta-epidemiological studies (2). In this review, statistically significantly larger effect estimates were established for trials with <100 patients per intervention group (overestimation = 33%) (15, 16), for lack of double blinding (overestimation = 9 and 13%) (17, 18) and lack of allocation concealment (dSMD 0.15; 95%CI: 0.03 to 0.28; *I*^2^ = 0%) (19, 20).

### 4.2 Study limitations

Due to its exploratory nature, our study has several shortcomings that should be addressed in further research. Our study only compared the novel CQS-2B against Cochrane’s RoB 2 tool as the current excepted gold standard and only used one single systematic review for comparison. Albeit we deem our study design sufficient for first-time exploration of the topic and hypothesis generation, its anecdotal nature is insufficient to draw broader conclusions. Particularly, our study is unable to answer the question whether the use of the CQS-2B yields more conservative conclusions than other clinical trial appraisal tools, in general, apart from the RoB2. For this, further testing of the CQS-2B in comparison with other appraisal tools is necessary. Furthermore, its application in systematic reviews that include trials with corroboration level 4 may show no more conservative conclusions than based on the RoB2 tool.

### 4.3 Hypothesis development and recommendations for further research

Mickenautsch et al. (21) have only recently developed the Composite Quality Score (CQS) and thus the CQS in its latest version (CQS-2B) is still a novel trial appraisal tool to date. The tool is also unique in its reliance on the deductive-falsification approach (1) and therefore its applicability and validity have not previously been studied in depth.

Within this context, our current study was the first to explore whether conclusions from a systematic review that applied the second version of Cochrane’s RoB 2 tool (as the current gold standard for the appraisal of prospective, controlled, clinical therapy trials (10)) do not differ when the CQS-2B is used, instead. The current results are the first available evidence to the topic and support the hypothesis:

**Trial appraisal using the CQS-2B provides a basis for more conservative systematic review conclusions that are more sensitive to potential bias risk than trial appraisal using Cochrane’s RoB 2 tool.**

This hypothesis is amendable for testing, ideally on the basis of data from a number of systematic reviews that are randomly selected from several databases with relevance to clinical therapy research, such as PubMed and Embase. The appropriate number of systematic reviews should be established on the basis of sample size calculation and should cover a wide range of fields of study that are related to clinical therapy. Systematic review selection should not be limited (as was the case in this study) to open access reports with English as the only publication language. It may further be of advantage that the selected systematic review reports include stratification by overall risk of bias for single outcomes or endpoints as recommended by Sterne et al. (11). Contrary to such recommendation, stratification was not conducted by the authors of our selected systematic review (14) and thus we had to conduct it for our study ourselves.

It may further be of advantage that reviewers who apply the CQS-2B also re-appraise trials using the RoB 2 tool and not rely only on the overall bias risk reported in the systematic reviews. Measurement error between RoB 2 and CQS-2B rating may thus be reduced when the same reviewers apply both tools, particularly against the background of the RoB 2 – tool’s low inter-rater reliability (8).

Hypothesis testing should include a two-step approach. (i) Statistical testing of the null-hypothesis that pooled effect estimates from trials with the highest corroboration level with data, rated according to the CQS-2B criteria, does not significantly differ from trials of lowest bias risk, rated according to the RoB 2 tool, with the significance level set at 5%. (ii) Investigation whether conclusions from the established effect estimates are more conservative when in line with the CQS2B than with the RoB 2 tool. The current hypothesis will be falsified if either the null-hypothesis is not accepted, the conclusions from the established effect estimates are not more conservative when in line with the CQS2B or when both are the case.

## 5 Conclusion

The results of this exploratory study provided the basis for the generation of the hypothesis that trial appraisal using the CQS-2B provides a basis for more conservative systematic review conclusions that are more sensitive to potential bias risk than trial appraisal using Cochrane’s RoB 2 tool. This hypothesis is amenable to future testing, specifically on basis of trial data from a random sample of systematic review reports.

## Data availability statement

The original contributions presented in the study are included in the article/Supplementary material, further inquiries can be directed to the corresponding author.

## Author contributions

SM: Conceptualization, Data curation, Formal analysis, Investigation, Methodology, Project administration, Resources, Supervision, Validation, Visualization, Writing – original draft, Writing – review & editing. SR: Investigation, Validation, Writing – review & editing. VY: Resources, Validation, Writing – review & editing.
