# Supplementary material for: Application of the Composite Quality Score (CQS-2B) versus Cochrane’s Risk of Bias tool (Version 2) in systematic reviews of clinical trials – an exploratory study
Source: Front Med (Lausanne). 2024 May 2;11:1307815. doi: 10.3389/fmed.2024.1307815 (PMC11096475; doi:10.3389/fmed.2024.1307815)
Supplement: Supplementary file 3 [file Data_Sheet_1.PDF]

Application of the Composite Quality Score (CQS-2B) versus Cochrane's Risk of Bias tool (Version 2) in systematic reviews of clinical trials – An exploratory study

Steffen Mickenautsch, Stefan Rupf, Veerasamy Yengopal

<u>Comparison 1: Palmitic acid vs MUFAs+PUFAs</u>

RoB 2			CQS-2B		
Item		Remark	Item		Remark
Measured outcome 1: Effect of dietary fat substitutions on LDL-C					
[1] Total number of trials appraised:		18	[1] Total number of trials appraised:		18
[2] Trials with overall 'low bias risk'	[a] Number of trials:	3	[2] Trials with highest corroboration (C-) level	[a] C-level:	C2
	[b] Effect estimate (pooled if >1 trials):	MD –0.28 (95%CI: -0.81; 0.24)		[b] Number of trials:	1
				[c] Effect estimate (pooled if >1 trials):	MD –0.47 (95%CI: -0.82; -0.12)
[3] Clinically relevant conclusion based on [2.b]:		Replacement of palmitic acid with MUFAs+PUFAs appears to have **no beneficial effect** on the fasting LDL-cholesterol concentration.	[3] Clinically relevant conclusion based on [2.c] possible? (Yes/No):		No
			[4.a] If [3] 'Yes' - Clinically relevant conclusion based on [2.c] (from all trial results at C4 – level):		N/A
			[4.b] If [3] 'No' – Reason:		Falsification of the established effect estimate [2.c] at C3 level (Criterion III = 0-score), due to lack or insufficient reporting of double blinding, resulting in high risk that the estimate is overestimated and therefore may not reflect the true clinical effect on the fasting LDL-cholesterol concentration after substitution of palimitic acid with MUFAs+PUFAs.
Measured outcome 2: Effect of dietary fat substitutions on TC					
[1] Total number of trials appraised:		18	[1] Total number of trials appraised:		18
[2] Trials with overall 'low bias risk'	[a] Number of trials:	3	[2] Trials with highest corroboration (C-) level	[a] C-level:	C2
	[b] Effect estimate (pooled if >1 trials):	MD –0.48 (95%CI: -0.79; -0.18)		[b] Number of trials:	1
				[c] Effect estimate (pooled if >1 trials):	MD –0.60 (95%CI: -1.02; -0.18)
[3] Clinically relevant conclusion based on [2.b]:		Replacement of palmitic acid with MUFAs+PUFAs appears to have a **beneficial effect** on the fasting total cholesterol concentration.	[3] Clinically relevant conclusion based on [2.c] possible? (Yes/No):		No
			[4.a] If [3] 'Yes' - Clinically relevant conclusion based on [2.c] (from all trial results at C4 – level):		N/A
			[4.b] If [3] 'No' – Reason:		Falsification of the established effect estimate [2.c] at C3 level (Criterion III = 0-score), due to lack or insufficient reporting of double blinding, resulting in high risk that the estimate is overestimated and therefore may not reflect the true clinical effect on the fasting total cholesterol concentration after substitution of palimitic acid with MUFAs+PUFAs.

RoB 2			CQS-2B		
Item		Remark	Item		Remark
Measured outcome 3: Effect of dietary fat substitutions on HDL-C					
[1] Total number of trials appraised:		18	[1] Total number of trials appraised:		18
[2] Trials with overall 'low bias risk'	[a] Number of trials:	3	[2] Trials with highest corroboration (C-) level	[a] C-level:	C2
	[b] Effect estimate (pooled if >1 trials):	MD –0.10 (95%CI: -0.19; -0.02)		[b] Number of trials:	1
				[c] Effect estimate (pooled if >1 trials):	MD –0.12 (95%CI: -0.28; 0.04)
[3] Clinically relevant conclusion based on [2.b]:		Replacement of palmitic acid with MUFAs+PUFAs appears to have a **beneficial effect** on the fasting HDL cholesterol concentration.	[3] Clinically relevant conclusion based on [2.c] possible? (Yes/No):		No
			[4.a] If [3] 'Yes' - Clinically relevant conclusion based on [2.c] (from all trial results at C4 – level):		N/A
			[4.b] If [3] 'No' – Reason:		Falsification of the established effect estimate [2.c] at C3 level (Criterion III = 0-score), due to lack or insufficient reporting of double blinding, resulting in high risk that the estimate is overestimated and therefore may not reflect the true clinical effect on the fasting HDL-cholesterol concentration after substitution of palimitic acid with MUFAs+PUFAs.
Measured outcome 4: Effect of dietary fat substitutions on triacylglycerol					
[1] Total number of trials appraised:		18	[1] Total number of trials appraised:		18
[2] Trials with overall 'low bias risk'	[a] Number of trials:	3	[2] Trials with highest corroboration (C-) level	[a] C-level:	C2
	[b] Effect estimate (pooled if >1 trials):	MD 0.01 (95%CI: -0.16; 0.17)		[b] Number of trials:	1
				[c] Effect estimate (pooled if >1 trials):	MD –0.04 (95%CI: -0.29; -0.21)
[3] Clinically relevant conclusion based on [2.b]:		Replacement of palmitic acid with MUFAs+PUFAs appears to have **no beneficial effect** on the fasting triacylglycerol concentration.	[3] Clinically relevant conclusion based on [2.c] possible? (Yes/No):		No
			[4.a] If [3] 'Yes' - Clinically relevant conclusion based on [2.c] (from all trial results at C4 – level):		N/A
			[4.b] If [3] 'No' – Reason:		Falsification of the established effect estimate [2.c] at C3 level (Criterion III = 0-score), due to lack or insufficient reporting of double blinding, resulting in high risk that the estimate is overestimated and therefore may not reflect the true clinical effect on the fasting triacylglycerol concentration after substitution of palimitic acid with MUFAs+PUFAs.

RoB 2			CQS-2B		
Item		Remark	Item		Remark
Measured outcome 5: Effect of dietary fat substitutions on apoA-I concentrations					
[1] Total number of trials appraised:		18	[1] Total number of trials appraised:		18
[2] Trials with overall 'low bias risk'	[a] Number of trials:	3	[2] Trials with highest corroboration (C-) level	[a] C-level:	C2
	[b] Effect estimate (pooled if >1 trials):	MD –0.06 (95%CI: -0.15; 0.03)		[b] Number of trials:	1
				[c] Effect estimate (pooled if >1 trials):	MD –0.12 (95%CI: -0.25; 0.01)
[3] Clinically relevant conclusion based on [2.b]:		Replacement of palmitic acid with MUFAs+PUFAs appears to have **no beneficial effect** on the fasting apoA-I concentration.	[3] Clinically relevant conclusion based on [2.c] possible? (Yes/No):		No
			[4.a] If [3] 'Yes' - Clinically relevant conclusion based on [2.c] (from all trial results at C4 – level):		N/A
			[4.b] If [3] 'No' – Reason:		Falsification of the established effect estimate [2.c] at C3 level (Criterion III = 0-score), due to lack or insufficient reporting of double blinding, resulting in high risk that the estimate is overestimated and therefore may not reflect the true clinical effect on the fasting apoA-I concentration after substitution of palimitic acid with MUFAs+PUFAs.
Measured outcome 6: Effect of dietary fat substitutions on apoB concentrations					
[1] Total number of trials appraised:		18	[1] Total number of trials appraised:		18
[2] Trials with overall 'low bias risk'	[a] Number of trials:	3	[2] Trials with highest corroboration (C-) level	[a] C-level:	C2
	[b] Effect estimate (pooled if >1 trials):	MD –0.08 (95%CI: -0.15; -0.02)		[b] Number of trials:	1
				[c] Effect estimate (pooled if >1 trials):	MD –0.08 (95%CI: -0.17; 0.01)
[3] Clinically relevant conclusion based on [2.b]:		Replacement of palmitic acid with MUFAs+PUFAs appears to have a **beneficial effect** on the fasting apoB concentration.	[3] Clinically relevant conclusion based on [2.c] possible? (Yes/No):		No
			[4.a] If [3] 'Yes' - Clinically relevant conclusion based on [2.c] (from all trial results at C4 – level):		N/A
			[4.b] If [3] 'No' – Reason:		Falsification of the established effect estimate [2.c] at C3 level (Criterion III = 0-score), due to lack or insufficient reporting of double blinding, resulting in high risk that the estimate is overestimated and therefore may not reflect the true clinical effect on the fasting apoB concentration after substitution of palimitic acid with MUFAs+PUFAs.

Comparison 2: Palmitic acid vs Stearic acid

RoB 2			CQS-2B		
Item		Remark	Item		Remark
Measured outcome 1: Effect of dietary fat substitutions on LDL-C					
[1] Total number of trials appraised:		5	[1] Total number of trials appraised:		5
[2] Trials with overall ‘low bias risk’	[a] Number of trials:	3	[2] Trials with highest corroboration (C-) level	[a] C-level:	C3
	[b] Effect estimate (pooled if >1 trials):	MD –0.03 (95%CI: -0.30; 0.25)		[b] Number of trials:	1
				[c] Effect estimate (pooled if >1 trials):	MD –0.14 (95%CI: -0.70; 0.42)
[3] Clinically relevant conclusion based on [2.b]:		Replacement of palmitic acid with stearic acid appears to have **no beneficial effect** on the fasting LDL-cholesterol concentration.	[3] Clinically relevant conclusion based on [2.c] possible? (Yes/No):		No
			[4.a] If [3] ‘Yes’ - Clinically relevant conclusion based on [2.c] (from all trial results at C4 – level):		N/A
			[4.b] If [3] ‘No’ – Reason:		Falsification of the established effect estimate [2.c] at C4 level (Criterion IV = 0-score), due insufficient sample size, resulting in high risk that the estimate is overestimated and therefore may not reflect the true clinical effect on the fasting LDL-cholesterol concentration after substitution of palimitic acid with stearic acid.
Measured outcome 2: Effect of dietary fat substitutions on TC					
[1] Total number of trials appraised:		5	[1] Total number of trials appraised:		5
[2] Trials with overall ‘low bias risk’	[a] Number of trials:	3	[2] Trials with highest corroboration (C-) level	[a] C-level:	C3
	[b] Effect estimate (pooled if >1 trials):	MD –0.10 (95%CI: -0.42; 0.21)		[b] Number of trials:	1
				[c] Effect estimate (pooled if >1 trials):	MD –0.19 (95%CI: -0.79; 0.41)
[3] Clinically relevant conclusion based on [2.b]:		Replacement of palmitic acid with stearic acid appears to have **no beneficial effect** on the fasting total cholesterol concentration.	[3] Clinically relevant conclusion based on [2.c] possible? (Yes/No):		No
			[4.a] If [3] ‘Yes’ - Clinically relevant conclusion based on [2.c] (from all trial results at C4 – level):		N/A
			[4.b] If [3] ‘No’ – Reason:		Falsification of the established effect estimate [2.c] at C4 level (Criterion IV = 0-score), due to insufficient sample size, resulting in high risk that the estimate is overestimated and therefore may not reflect the true clinical effect on the fasting total cholesterol concentration after substitution of palimitic acid with stearic acid.

<table>
<tr><td colspan="3">RoB 2</td><td colspan="3">CQS-2B</td></tr>
<tr><td colspan="2">Item</td><td>Remark</td><td colspan="2">Item</td><td>Remark</td></tr>
<tr><td colspan="6">Measured outcome 3: Effect of dietary fat substitutions on HDL-C</td></tr>
<tr><td colspan="2">[1] Total number of trials appraised:</td><td>5</td><td colspan="2">[1] Total number of trials appraised:</td><td>5</td></tr>
<tr><td rowspan="3">[2] Trials with overall 'low bias risk'</td><td>[a] Number of trials:</td><td>3</td><td rowspan="3">[2] Trials with highest corroboration (C-) level</td><td>[a] C-level:</td><td>C3</td></tr>
<tr><td rowspan="2">[b] Effect estimate (pooled if >1 trials):</td><td rowspan="2">MD –0.06 (95%CI: -0.15; 0.04)</td><td>[b] Number of trials:</td><td>1</td></tr>
<tr><td>[c] Effect estimate (pooled if >1 trials):</td><td>MD –0.09 (95%CI: -0.22; 0.04)</td></tr>
<tr><td colspan="2" rowspan="3">[3] Clinically relevant conclusion based on [2.b]:</td><td rowspan="3">Replacement of palmitic acid with stearic acid appears to have no beneficial effect on the fasting HDL- cholesterol concentration.</td><td colspan="2">[3] Clinically relevant conclusion based on [2.c] possible? (Yes/No):</td><td>No</td></tr>
<tr><td colspan="2">[4.a] If [3] 'Yes' - Clinically relevant conclusion based on [2.c] (from all trial results at C4 – level):</td><td>N/A</td></tr>
<tr><td colspan="2">[4.b] If [3] 'No' – Reason:</td><td>Falsification of the established effect estimate [2.c] at C4 level (Criterion IV = 0-score), due to insufficient sample size, resulting in high risk that the estimate is overestimated and therefore may not reflect the true clinical effect on the fasting HDL- cholesterol concentration after substitution of palimitic acid with stearic acid.</td></tr>
<tr><td colspan="6">Measured outcome 4: Effect of dietary fat substitutions on triacylglycerol</td></tr>
<tr><td colspan="2">[1] Total number of trials appraised:</td><td>5</td><td colspan="2">[1] Total number of trials appraised:</td><td>5</td></tr>
<tr><td rowspan="3">[2] Trials with overall 'low bias risk'</td><td>[a] Number of trials:</td><td>3</td><td rowspan="3">[2] Trials with highest corroboration (C-) level</td><td>[a] C-level:</td><td>C3</td></tr>
<tr><td rowspan="2">[b] Effect estimate (pooled if >1 trials):</td><td rowspan="2">MD –0.05 (95%CI: -0.25; 0.15)</td><td>[b] Number of trials:</td><td>1</td></tr>
<tr><td>[c] Effect estimate (pooled if >1 trials):</td><td>MD –0.08 (95%CI: -0.20; 0.36)</td></tr>
<tr><td colspan="2" rowspan="3">[3] Clinically relevant conclusion based on [2.b]:</td><td rowspan="3">Replacement of palmitic acid with stearic acid appears to have no beneficial effect on the fasting triacylglycerol concentration.</td><td colspan="2">[3] Clinically relevant conclusion based on [2.c] possible? (Yes/No):</td><td>No</td></tr>
<tr><td colspan="2">[4.a] If [3] 'Yes' - Clinically relevant conclusion based on [2.c] (from all trial results at C4 – level):</td><td>N/A</td></tr>
<tr><td colspan="2">[4.b] If [3] 'No' – Reason:</td><td>Falsification of the established effect estimate [2.c] at C4 level (Criterion IV = 0-score), due to lack of insufficient sample size, resulting in high risk that the estimate is overestimated and therefore may not reflect the true clinical effect on the fasting triacylglycerol concentration after substitution of palimitic acid with stearic acid.</td></tr>
</table>

<u>Comparison 3: Palmitic acid vs Oleic acid</u>

<table>
<tr><td colspan="3">RoB 2</td><td colspan="3">CQS-2B</td></tr>
<tr><td colspan="2">Item</td><td>Remark</td><td colspan="2">Item</td><td>Remark</td></tr>
<tr><td colspan="6">Measured outcome 1: Effect of dietary fat substitutions on LDL-C</td></tr>
<tr><td colspan="2">[1] Total number of trials appraised:</td><td>9</td><td colspan="2">[1] Total number of trials appraised:</td><td>9</td></tr>
<tr><td rowspan="3">[2] Trials with overall 'low bias risk'</td><td>[a] Number of trials:</td><td>3</td><td rowspan="3">[2] Trials with highest corroboration (C-) level</td><td>[a] C-level:</td><td>C1</td></tr>
<tr><td rowspan="2">[b] Effect estimate (pooled if >1 trials):</td><td rowspan="2">MD –0.09 (95%CI: -0.36; 0.18)</td><td>[b] Number of trials:</td><td>9</td></tr>
<tr><td>[c] Effect estimate (pooled if >1 trials):</td><td>MD –0.16 (95%CI: -0.25; -0.06)</td></tr>
<tr><td colspan="2" rowspan="3">[3] Clinically relevant conclusion based on [2.b]:</td><td rowspan="3">Replacement of palmitic acid with oleic acid appears to have a no beneficial effect on the fasting LDL-cholesterol concentration.</td><td colspan="2">[3] Clinically relevant conclusion based on [2.c] possible? (Yes/No):</td><td>No</td></tr>
<tr><td colspan="2">[4.a] If [3] 'Yes' - Clinically relevant conclusion based on [2.c] (from all trial results at C4 – level):</td><td>N/A</td></tr>
<tr><td colspan="2">[4.b] If [3] 'No' – Reason:</td><td>Falsification of the established effect estimate [2.c] at C2 level (Criterion II = 0-score), due to lack or insufficient reporting of allocation concealment, resulting in high risk that the estimate is overestimated and therefore may not reflect the true clinical effect on the fasting LDL-cholesterol concentration after substitution of palimitic acid with oleic acid.</td></tr>
<tr><td colspan="6">Measured outcome 2: Effect of dietary fat substitutions on TC</td></tr>
<tr><td colspan="2">[1] Total number of trials appraised:</td><td>9</td><td colspan="2">[1] Total number of trials appraised:</td><td>9</td></tr>
<tr><td rowspan="3">[2] Trials with overall 'low bias risk'</td><td>[a] Number of trials:</td><td>3</td><td rowspan="3">[2] Trials with highest corroboration (C-) level</td><td>[a] C-level:</td><td>C1</td></tr>
<tr><td rowspan="2">[b] Effect estimate (pooled if >1 trials):</td><td rowspan="2">MD –0.18 (95%CI: -0.46; 0.10)</td><td>[b] Number of trials:</td><td>9</td></tr>
<tr><td>[c] Effect estimate (pooled if >1 trials):</td><td>MD –0.22 (95%CI: -0.34; 0.11)</td></tr>
<tr><td colspan="2" rowspan="3">[3] Clinically relevant conclusion based on [2.b]:</td><td rowspan="3">Replacement of palmitic acid with oleic acid appears to have a no beneficial effect on the fasting total cholesterol concentration.</td><td colspan="2">[3] Clinically relevant conclusion based on [2.c] possible? (Yes/No):</td><td>No</td></tr>
<tr><td colspan="2">[4.a] If [3] 'Yes' - Clinically relevant conclusion based on [2.c] (from all trial results at C4 – level):</td><td>N/A</td></tr>
<tr><td colspan="2">[4.b] If [3] 'No' – Reason:</td><td>Falsification of the established effect estimate [2.c] at C2 level (Criterion II = 0-score), due to lack or insufficient reporting of allocation concealment, resulting in high risk that the estimate is overestimated and therefore may not reflect the true clinical effect on the fasting total cholesterol concentration after substitution of palimitic acid with oleic acid.</td></tr>
</table>

<table>
<tr><td colspan="3">RoB 2</td><td colspan="3">CQS-2B</td></tr>
<tr><td colspan="2">Item</td><td>Remark</td><td colspan="2">Item</td><td>Remark</td></tr>
<tr><td colspan="6">Measured outcome 3: Effect of dietary fat substitutions on HDL-C</td></tr>
<tr><td colspan="2">[1] Total number of trials appraised:</td><td>9</td><td colspan="2">[1] Total number of trials appraised:</td><td>9</td></tr>
<tr><td rowspan="3">[2] Trials with overall 'low bias risk'</td><td>[a] Number of trials:</td><td>3</td><td rowspan="3">[2] Trials with highest corroboration (C-) level</td><td>[a] C-level:</td><td>C1</td></tr>
<tr><td rowspan="2">[b] Effect estimate (pooled if >1 trials):</td><td rowspan="2">MD –0.05 (95%CI: -0.13; 0.03)</td><td>[b] Number of trials:</td><td>9</td></tr>
<tr><td>[c] Effect estimate (pooled if >1 trials):</td><td>MD –0.04 (95%CI: -0.07; 0.00)</td></tr>
<tr><td colspan="2" rowspan="3">[3] Clinically relevant conclusion based on [2.b]:</td><td rowspan="3">Replacement of palmitic acid with oleic acid appears to have a no beneficial effect on the fasting HDL- cholesterol concentration.</td><td colspan="2">[3] Clinically relevant conclusion based on [2.c] possible? (Yes/No):</td><td>No</td></tr>
<tr><td colspan="2">[4.a] If [3] 'Yes' - Clinically relevant conclusion based on [2.c] (from all trial results at C4 – level):</td><td>N/A</td></tr>
<tr><td colspan="2">[4.b] If [3] 'No' – Reason:</td><td>Falsification of the established effect estimate [2.c] at C2 level (Criterion II = 0-score), due to lack or insufficient reporting of allocation concealment, resulting in high risk that the estimate is overestimated and therefore may not reflect the true clinical effect on the fasting HDL- cholesterol concentration after substitution of palimitic acid with oleic acid.</td></tr>
<tr><td colspan="6">Measured outcome 4: Effect of dietary fat substitutions on triacylglycerol</td></tr>
<tr><td colspan="2">[1] Total number of trials appraised:</td><td>9</td><td colspan="2">[1] Total number of trials appraised:</td><td>9</td></tr>
<tr><td rowspan="3">[2] Trials with overall 'low bias risk'</td><td>[a] Number of trials:</td><td>3</td><td rowspan="3">[2] Trials with highest corroboration (C-) level</td><td>[a] C-level:</td><td>C1</td></tr>
<tr><td rowspan="2">[b] Effect estimate (pooled if >1 trials):</td><td rowspan="2">MD –0.10 (95%CI: -0.23; 0.04)</td><td>[b] Number of trials:</td><td>9</td></tr>
<tr><td>[c] Effect estimate (pooled if >1 trials):</td><td>MD 0.01 (95%CI: -0.07; 0.09)</td></tr>
<tr><td colspan="2" rowspan="3">[3] Clinically relevant conclusion based on [2.b]:</td><td rowspan="3">Replacement of palmitic acid with oleic acid appears to have a no beneficial effect on the fasting triacylglycerol concentration.</td><td colspan="2">[3] Clinically relevant conclusion based on [2.c] possible? (Yes/No):</td><td>No</td></tr>
<tr><td colspan="2">[4.a] If [3] 'Yes' - Clinically relevant conclusion based on [2.c] (from all trial results at C4 – level):</td><td>N/A</td></tr>
<tr><td colspan="2">[4.b] If [3] 'No' – Reason:</td><td>Falsification of the established effect estimate [2.c] at C2 level (Criterion II = 0-score), due to lack or insufficient reporting of allocation concealment, resulting in high risk that the estimate is overestimated and therefore may not reflect the true clinical effect on the fasting triacylglycerol concentration after substitution of palimitic acid with oleic acid.</td></tr>
</table>

RoB 2			CQS-2B		
Item		Remark	Item		Remark
Measured outcome 5: Effect of dietary fat substitutions on apoA-I concentrations					
[1] Total number of trials appraised:		5	[1] Total number of trials appraised:		5
[2] Trials with overall 'low bias risk'	[a] Number of trials:	1	[2] Trials with highest corroboration (C-) level	[a] C-level:	C1
	[b] Effect estimate (pooled if >1 trials):	MD 0.04 (95%CI: -0.09; 0.17)		[b] Number of trials:	5
				[c] Effect estimate (pooled if >1 trials):	MD -0.01 (95%CI: -0.06; 0.03)
[3] Clinically relevant conclusion based on [2.b]:		Replacement of palmitic acid with oleic acid appears to have a **no beneficial effect** on the fasting apoA-I concentration.	[3] Clinically relevant conclusion based on [2.c] possible? (Yes/No):		No
			[4.a] If [3] 'Yes' - Clinically relevant conclusion based on [2.c] (from all trial results at C4 – level):		N/A
			[4.b] If [3] 'No' – Reason:		Falsification of the established effect estimate [2.c] at C2 level (Criterion II = 0-score), due to lack or insufficient reporting of allocation concealment, resulting in high risk that the estimate is overestimated and therefore may not reflect the true clinical effect on the fasting apoA-I concentration after substitution of palimitic acid with oleic acid.
Measured outcome 6: Effect of dietary fat substitutions on apoB concentrations					
[1] Total number of trials appraised:		5	[1] Total number of trials appraised:		5
[2] Trials with overall 'low bias risk'	[a] Number of trials:	1	[2] Trials with highest corroboration (C-) level	[a] C-level:	C1
	[b] Effect estimate (pooled if >1 trials):	MD -0.04 (95%CI: -0.15; 0.07)		[b] Number of trials:	5
				[c] Effect estimate (pooled if >1 trials):	MD -0.05 (95%CI: -0.09; 0.00)
[3] Clinically relevant conclusion based on [2.b]:		Replacement of palmitic acid with oleic acid appears to have a **no beneficial effect** on the fasting apoB concentration.	[3] Clinically relevant conclusion based on [2.c] possible? (Yes/No):		No
			[4.a] If [3] 'Yes' - Clinically relevant conclusion based on [2.c] (from all trial results at C4 – level):		N/A
			[4.b] If [3] 'No' – Reason:		Falsification of the established effect estimate [2.c] at C2 level (Criterion II = 0-score), due to lack or insufficient reporting of allocation concealment, resulting in high risk that the estimate is overestimated and therefore may not reflect the true clinical effect on the fasting apoB concentration after substitution of palimitic acid with oleic acid.

SD = Standard deviation
CI = Confidence interval
PUFA/MUFA = monounsaturated/polyunsaturated fatty acid
LDL-C = LDL cholesterol
HDL-C = cholesterol
TC = total cholesterol
C1-4 level = Corroboration level 1-4
N = Number of subjects per trial group
MD = Mean difference
N/A = Not applicable
